# Acute effects of static balance exercise combined with different levels of blood flow restriction on motor performance fatigue as well as physiological and perceptual responses in young healthy males and females

**DOI:** 10.1007/s00421-023-05258-5

**Published:** 2023-07-11

**Authors:** Robert Bielitzki, Tom Behrendt, Andy Weinreich, Thomas Mittlmeier, Lutz Schega, Martin Behrens

**Affiliations:** 1 Department of Sport Science, Institute III, Otto-von-Guericke University Magdeburg, 39104 Magdeburg, Germany; 2 Department of Traumatology, Hand-and Reconstructive Surgery, Rostock University Medical Center, Schillingallee 35, 18057 Rostock, Germany

**Keywords:** Vascular occlusion, Effort, Pain, Muscle activity, Fatigability, Recovery, Postural control

## Abstract

**Purpose:**

This study investigated the acute effects of a static balance exercise combined with different blood flow restriction (BFR) pressures on motor performance fatigue development and recovery as well as physiological and perceptual responses during exercise in males and females.

**Methods:**

Twenty-four recreational active males (n = 13) and females (n = 11) performed static balance exercise on a BOSU ball (3 sets of 60 s with 30 s rest in-between) on three separate (> 3 days) laboratory visits with three different BFR pressures (80% arterial occlusion pressure [AOP], 40%AOP, 30 mmHg [SHAM]) in random order. During exercise, activity of various leg muscles, vastus lateralis muscle oxygenation, and ratings of effort and pain perception were recorded. Maximal squat jump height was measured before, immediately after, 1, 2, 4, and 8 min after exercise to quantify motor performance fatigue development and recovery.

**Results:**

Quadriceps muscle activity as well as ratings of effort and pain were highest, while muscle oxygenation was lowest in the 80%AOP compared to the 40%AOP and SHAM condition, with no differences in postural sway between conditions. Squat jump height declined after exercise with the highest reduction in the 80%AOP (− 16.4 ± 5.2%) followed by the 40%AOP (− 9.1 ± 3.2%), and SHAM condition (− 5.4 ± 3.3%). Motor performance fatigue was not different after 1 min and 2 min of recovery in 40% AOP and 80% AOP compared to SHAM, respectively.

**Conclusion:**

Static balance exercise combined with a high BFR pressure induced the largest changes in physiological and perceptual responses, without affecting balance performance. Although motor performance fatigue was increased by BFR, it may not lead to long-term impairments in maximal performance.

**Supplementary Information:**

The online version contains supplementary material available at 10.1007/s00421-023-05258-5.

## Introduction

Blood flow restriction (BFR) training is characterized by the application of inflatable cuffs, non-pneumatic elastic wraps or rigid nylon straps at the proximal portion of a limb during exercise in order to reduce arterial inflow and block venous return of blood in the respective extremity (Bielitzki et al. 2021). Its application is assumed to amplify intramuscular hypoxia (Biazon et al. 2019) (also known as localized hypoxia) distal to the cuff as a result of an inadequate oxygen supply and blood pooling (Jessee et al. 2018). Although the exact physiological mechanisms triggered by BFR training are not fully understood, it is thought that the external manipulation of blood flow leads to an increased level of metabolic stress (e.g., accumulation of inorganic phosphate) (Pearson and Hussain 2015). Particularly, metabolic stress is theorized to be an important factor for the BFR-induced adaptations by acting on secondary factors, such as increased recruitment of muscle fibres (especially type II muscle fibres), probably due to an accelerated motor performance fatigue development (Husmann et al. 2018) (i.e., reduction in motor performance due to impairments in muscle activation and/or contractile function (Behrens et al. 2023)) and an increased hydration-mediated cell swelling (Pearson and Hussain 2015). It is widely accepted that metabolic stress, among other factors, can promote muscle hypertrophy (Wackerhage et al. 2019). In this context, it has been demonstrated that low-load resistance training combined with BFR produces greater increases in the relative deoxyhemoglobin concentration (i.e., a proxy marker of metabolic stress) compared to high-load (Biazon et al. 2019) and low-load (Lauver et al. 2017) resistance training without BFR. Furthermore, changes in the relative deoxyhemoglobin concentration were strongly associated (*r* = 0.716, *p* < 0.001) with alterations in muscle cross-sectional area after 10 weeks of low-load resistance training in combination with BFR (Biazon et al. 2019). Accordingly, there is growing evidence that low-load resistance training (e.g., 20–30% of the one repetition maximum [1RM]) combined with BFR induces increases in muscle mass and strength.

Additionally, exercise combined with BFR has been shown to promote the release of signalling proteins responsible for angiogenesis, mitochondrial biogenesis, increased blood flow, and anti-oxidant function, which could explain the improvements in measures of aerobic capacity (e.g., maximal or peak oxygen uptake) and endurance performance (e.g., six minute walk distance or time to exhaustion) reported in the literature (Bennett and Slattery 2019). Thereby, the extent of metabolic stress and the associated beneficial adaptations seem to depend on the cuff pressure (typically set between 40 and 80% of the arterial occlusion pressure (AOP)), with larger adaptations when using a higher AOP (Mouser et al. 2019).

So far, the acute effects of several exercise modalities combined with BFR (e.g., aerobic exercise (Hughes et al. 2021), resistance exercise (Husmann et al. 2018), and whole-body vibration (Centner et al. 2019)) on various outcomes have been investigated. However, to the best of our knowledge, the acute effects of balance exercise combined with BFR on physiological and perceptual responses have not yet been examined. Balance training has been shown to be an effective training strategy to improve measures of static and dynamic balance performance, for instance, in healthy older adults (Lesinski et al. 2015b). Interestingly, there is also evidence that balance training can increase maximal voluntary isometric contraction (MVIC) strength in recreationally active persons who engaged in physical training for 1.5 ± 3.0 years (Heitkamp et al. 2001).

According to the mechanisms triggered by BFR mentioned above (e.g., increased metabolic stress), it could be assumed that the addition of BFR to balance training may also lead to increases in training adaptations (e.g., muscle strength). Moreover, it was shown that performing balance training after the soccer training session (20 min per session, three times per week for 12 weeks), in a potentially fatigued state, increased balance performance in soccer players more than conducting the balance training before the soccer training session (Gioftsidou et al. 2006). Therefore, it might be that the hypothetically accelerated motor performance fatigue during balance exercise combined with BFR increases balance performance in the long-term. Nevertheless, given that chronic adaptations to training seem to strongly depend on the acute physiological responses (e.g., association of the acute changes in deoxyhemoglobin with the changes in muscle cross-sectional area (Biazon et al. 2019)), it is of particular importance to investigate the acute effects in order to optimize long-term training adaptations. Furthermore, assuming that balance training combined with BFR promotes motor performance fatigue development, as shown for low-load resistance training combined with BFR (Husmann et al. 2018), sex-related differences could be expected. There is evidence that females often exhibit a lower motor performance fatigue compared to males during single-joint isometric and slow to moderate velocity muscle actions as well as whole-body exercise (Hunter 2018; Ansdell et al. 2019, 2020). It is assumed that this is due to physiological differences between females and males, with females having a larger percentage of type I muscle fibres associated with a higher capillarization, mitochondrial respiratory capacity, and muscle perfusion as well as a lower glycogen utilization compared to males. These differences can result in a slower accumulation of metabolites, and, in turn, a slower decline in muscle activation and/or contractile function of muscles, which determine the extent of motor performance fatigue (Hunter 2014, 2018; Enoka and Duchateau 2016; Behrens et al. 2023).

Therefore, this study examined the acute effects of a static balance exercise combined with BFR on motor performance fatigue and recovery (i.e., changes in maximal squat jump height) as well as muscle activity, measures of metabolic stress (i.e., muscle oxygenation), balance performance (e.g., postural sway), and perceptual responses during exercise (i.e., ratings of effort perception and exercise-induced leg muscle pain). Furthermore, since the acute and chronic responses to BFR exercise or training, respectively, depend, among other factors, on the amount of the cuff pressure, two different individually adjusted cuff pressures (i.e., 40% and 80% of individuals’ AOP) were applied. We hypothesized that static balance exercise combined with BFR, and in particular with the high BFR pressure (80% AOP), will increase muscle activity, indices of metabolic stress, perceptual responses, as well as motor performance fatigue compared to static balance exercise without BFR (SHAM). Further, due to sex-related differences in the physiological adjustments during exercise and motor performance fatigue (Hunter 2014) as well as perceptual responses to exercise (Cook et al. 1998), we presumed lower physiological and perceptual changes in females compared to males.

## Material and methods

### Experimental procedure

All participants completed four laboratory visits consisting of a familiarization session and three randomized, counterbalanced experimental sessions with different conditions (i.e., 80% AOP, 40% AOP, and SHAM) in a cross-over design. Subjects were instructed to avoid consumption of alcohol, analgesics, and caffeine for 24 h and strenuous exercise for 48 h prior to the four laboratory visits. The sessions were separated by 5 ± 2 days and were conducted at the same time of day to reduce circadian variations.

During the first visit, the participants received written information about the experimental procedure and filled in two questionnaires (Physical Activity Readiness Questionnaire (German version), menstrual cycle questionnaire (only females)). Furthermore, participants’ (i) anthropometric data, (ii) systolic and diastolic blood pressure, and (iii) AOP were determined. Afterwards, participants were comprehensively familiarized with the (i) perceptual rating scales (ratings of effort perception and exercise-induced leg muscle pain), (ii) squat jump test, and (iii) two sets of static double-leg stance on an unstable surface followed by a single squat jump.

Upon arrival at the laboratory, the surface electrodes were applied to eight muscles of the right leg to record muscle activity during the experimental sessions. Participants performed three MVICs of the knee extensors, knee flexors, plantar flexors, and dorsiflexors to determine maximal muscle activity. Before (pre) and immediately after (post), as well as 1, 2, 4, and 8 min after completing the balance exercise in each condition, maximal squat jump height of the participants was determined to monitor motor performance fatigue development and recovery.

**Fig. 1:**
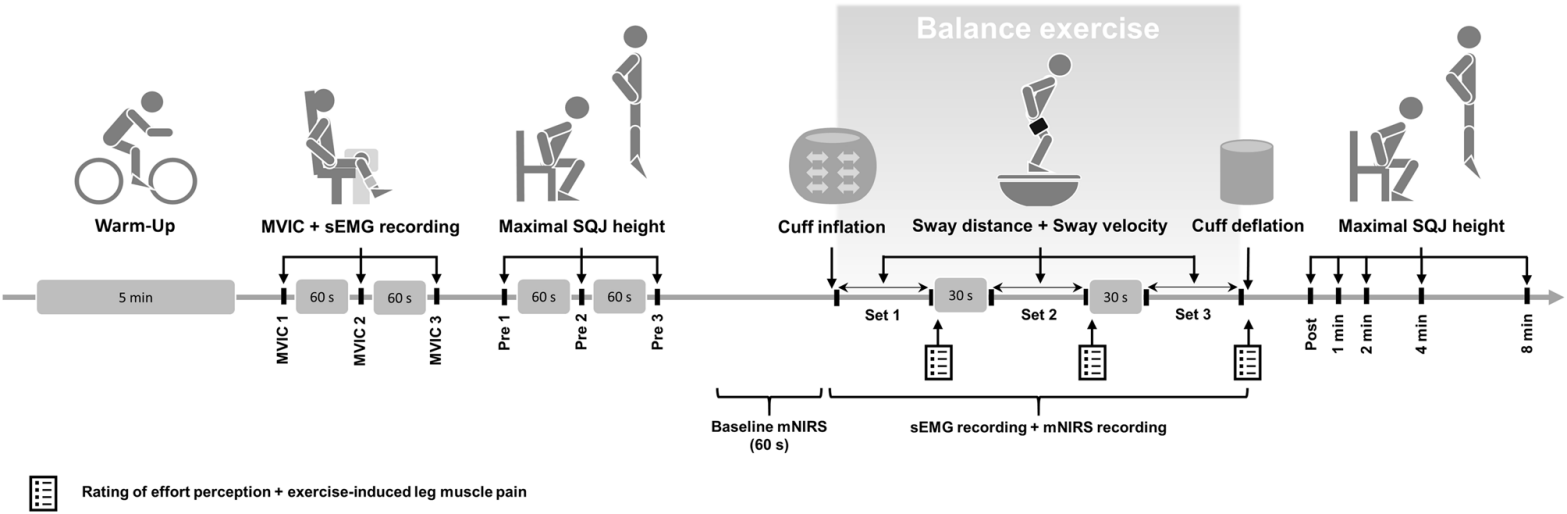
Schematic overview of the experimental procedure including warm-up, maximal voluntary isometric contractions (MVIC), squat jump height (SQJ) testing at pre, exercise protocol with surface electromyography (sEMG) and muscular near-infrared spectroscopy (mNIRS) recordings, as well as SQJ testing during the recovery phase

Surface electromyography (sEMG) was used to continuously record muscle activity of the quadriceps, hamstrings, triceps surae, and tibialis anterior during the balance exercise in each experimental condition. Muscle near-infrared spectroscopy (mNIRS) data were collected at the vastus lateralis of the right leg before (baseline) and during the balance exercise. Ratings of effort perception and exercise-induced leg muscle pain were recorded immediately after each set. The entire experimental protocol is shown in Fig. 1.

### Participants

A sample size calculation was conducted with the effect size of the time × condition interaction for muscle activity of the rectus femoris during the second set of whole-body vibration combined with BFR (Centner et al. 2019). The calculation for a 2 × 3 repeated measures ANOVA with a high effect size of *η*_*p*_^*2*^ = 0.200 (α = 0.05, 1–β = 0.95) revealed that a total sample size of 18 subjects would be required. Considering a drop-out rate of 20%, 22 participants had to be recruited. Therefore, 24 recreationally active subjects (males: *n* = 13, females: *n* = 11) voluntarily participated in the present study. Participants’ anthropometric characteristics are presented in Table 1. Exclusion criteria were defined as follows: (i) hypertension (> 140/90 mmHg), (ii) musculoskeletal injuries, (iii) neurological, mental, or cardiovascular disorders or diseases, (iv) medication with central nervous or cardiovascular effects, (v) pregnancy, (vi) open wounds or sensitive scar tissue at the lower limbs, and (vii) skinfold thickness of > 25 mm over the vastus lateralis (necessary for optimal mNIRS recordings according to manufacturer´s manual). Participants gave their written informed consent and the study was approved by the Ethics Committee of the Otto von Guericke University Magdeburg conforming to the principles of the Declaration of Helsinki on human experimentation.

**Table 1:** Participants’ characteristics

	N = 24 (11 f/13 m)
Age [years]	23.8 ± 3.1
Weight [kg]	67.0 ± 12.3
Height [cm]	174.3 ± 9.3
Body Mass Index [kg . m^−2^]	21.9 ± 2.5
Physical training [h . week^−1^]	7.2 ± 3.6
Systolic blood pressure [mmHg]	122.0 ± 8.7
Diastolic blood pressure [mmHg]	79.9 ± 4.7
Arterial occlusion pressure [mmHg]	223.5 ± 20.9
80% of arterial occlusion pressure [mmHg]	178.8 ± 16.7
40% of arterial occlusion pressure [mmHg]	89.4 ± 8.4

*f* female; *m* male

### Menstrual cycle

Female subjects were asked to provide the onset and duration of their previous menstrual cycle as well as the usage of oral contraceptives. According to Iacovides et al. (2015), the menstrual cycle was divided into four phases: (i) days 1–5 (menstruation), (ii) days 6–13 (follicular phase), (iii) day 14 (ovulation), and (iv) days 15–28 (luteal phase).

### Determination of BFR pressure

The individual AOP of the right leg was determined in the first session. Participants were seated in an upright position with their right foot on a box to achieve a hip, knee, and ankle joint angle of 90°. Participants rested for 10 min before a 10 × 76 cm pneumatic cuff (UT 1330-L, Ulrich Medical, Ulm, Germany) was applied to the most proximal part of the right thigh and connected to a medical tourniquet system (HeidiTM, Ulrich Medical, Ulm, Germany). A handheld, bidirectional, and highly sensitive 8 MHz Doppler probe (Dopplex DMX, Huntleigh Healthcare Ltd, Cardiff, UK) was placed over the posterior tibial artery with an insonation angle of approximately 45° opposite to the direction of flow according to the manufacturer’s manual to assess arterial blood flow. The cuff was progressively inflated until the blood flow could no longer be detected. The inflation protocol was conducted according to Loenneke et al. (2012). AOP was defined as the lowest cuff pressure at which arterial blood flow was not present (smallest increment = 10 mmHg). The AOP was used to apply an individualized cuff pressure for each participant during acute balance exercise (i.e., during the 80% and 40% AOP conditions). The same cuff pressure was applied to the right and left thigh as studies have shown no differences in AOP between legs (Hughes et al. 2021).

### Balance exercise protocol

The balance exercise protocol consisted of 3 sets of 60 s static double-leg stance on a BOSU ball (BOSU® Pro Balance Trainer, BOSU® Official Global Headquarters, Ashland, OH, USA) with 30 s rest between sets. The exercise normatives were in line with current recommendations for balance training (Lesinski et al. 2015a). During the first laboratory visit, participants were familiarized with the exercise sequences, the correct posture, and acoustic signals indicating start and stop of the exercise. The participants stood upright with a slightly inclined upper body, a knee angle between 30 and 45° (0° = full extension), and hands akimbo on the BOSU ball, which was placed on a force plate. The range of 15° in the knee angle was chosen because of the permanent adjustment of the posture due to the unstable surface. The knee angle was frequently checked using a goniometer on the participants’ left knee. During the balance task, participants should fix a point (16 mm diameter) at eye level 1 m in front of the force plate. The upper body and lower legs were approximately in line. The foot position on the BOSU ball was recorded in the first condition, to reproduce it in the subsequent test sessions. During the 30 s rest periods, the participants stayed on the BOSU ball and were instructed to relax their leg muscles by putting their weight on a walking aid in front of the force plate. The walking aid was also used to get safe on the BOSU ball. Acoustic signals were given for the start and end of each set. The BOSU ball was placed on the force plate in a standardized way and the pressure was kept constant during all conditions. The cuffs were applied to both thighs and inflated after the participants positioned themselves on the BOSU ball and immediately deflated after the last exercise set.

### Maximal squat jump height

Maximal squat jump height was quantified using a 1 m long optical sensor system (OptoGait, Microgate, Bolzano, Italy) and standardized instructions regarding the correct execution were given. Participants had to stand upright in the optical sensor system with their hands akimbo. Subsequently, they slowly bent their knees to reach the squat starting position, which was standardized and mechanically restricted with a chair (seat height = 70 cm) that had to be touched slightly with the buttocks. Participants maintained this position for ~ 2 s before performing a maximal squat jump. Prior to the three baseline squat jumps, the subjects performed a specific warm-up consisting of one set of ten squats and one set of five submaximal squat jumps with 2 min rest between sets.

Before each exercise condition, the maximal attempts were recorded with 1 min rest between each jump until the coefficient of variation of the best three trials was below 5%. Mean jump height of the three attempts was calculated and set as baseline for further data analyses. Immediately after the last static balance exercise set, participants executed another maximal squat jump to quantify motor performance fatigue (post). Thereafter, further maximal squat jumps were performed at 1, 2, 4, and 8 min post exercise, respectively, to determine the recovery of motor performance fatigue. The intraclass correlation coefficient revealed an excellent reliability for the pre-test maximal squat jump height between conditions (ICC_2,k_ = 0.994). For statistical analysis, data were quantified as the percentage changes of maximal squat jump height in relation to the baseline values to normalize the data.

### Balance performance

Balance performance was assessed using a force plate (NeuroCom Balance Manager®, Neuroswiss AG, Bern, Switzerland) measuring the ground reaction force during each set with a sampling frequency of 100 Hz. The trajectory of the center of pressure was processed using the MatLab software (MATLAB version R2020b, Math Works, Massachusetts, United States) to calculate the total sway distance, which represents the summed displacements in the medio-lateral and anterior–posterior direction, with the following formula:

$${\text{Total sway distance }} = \sum \sqrt {\left( {f\left( {x_{n} } \right) - f\left( {x_{n + 1} } \right)} \right)^{2} + \left( {f\left( {y_{n} } \right) - f\left( {y_{n + 1} } \right)} \right)^{2} }^{{}} .$$

The sway velocity was computed by the NeuroCom Balance Manager software and represents the ratio between the total distance covered by the center of pressure and the time of the sampled period. Prior to the testing sessions, the force plate was calibrated to the weight of the BOSU ball. Afterwards, the starting position was standardized by placing the BOSU ball in the middle of the force plate, which was verified using the center of pressure feedback function.

### Muscle activity

Muscle activity during balance exercise was measured using sEMG. The sEMG recording procedure was performed as described in detail by Behrens et al. (2015). Myoelectrical signals (Noraxon Desktop DTS, Noraxon U.S.A., Inc., Scottsdale, AZ, USA) of the vastus medialis, rectus femoris, vastus lateralis, semitendinosus/semimembranosus, biceps femoris, tibialis anterior, gastrocnemius medialis, and soleus were recorded using 30 × 24 mm gel-coated self-adhesive surface electrodes (Kendall ECG Electrodes, Covidien Ilc, Mansfield, MA, USA). The locations of the surface electrodes were marked to reproduce their application in the subsequent test sessions. The sEMG data recorded during the balance exercise were normalized to the maximal muscle activity obtained during MVIC for each muscle using a Biodex dynamometer (Biodex System 4 Pro, Biodex Medical Systems, Inc., New York, USA). The seating position was individually adjusted for each participant and settings were recorded for subsequent visits. Before MVIC testing, participants performed a standardized warm-up consisting of 5 min low load cycling (90 rpm at 100 W for males and 80 W for females) followed by one isometric contraction at 50%, 80%, and 100% of the estimated MVIC for each muscle group. MVIC of the knee extensors and knee flexors (70° knee and hip angle) as well as plantar flexors and dorsiflexors (70° hip angle, 30° knee angle, and 70° ankle angle (0° = full extension)) was recorded in the given order. Prior to each trial, subjects were instructed to cross their arms in front of their chest and to push or pull as fast and hard as possible for 5 s. Standardized and strong verbal encouragement was provided to achieve maximal performance. Visual feedback of the torque-time curve and the maximal torque value were provided. The maximal trials were assessed until the coefficient of variation of the best three attempts was below 5% (Behrens et al. 2015).

The sEMG signals were sampled at a rate of 1000 Hz, band pass filtered (8–450 Hz), and rectified. The first and last second of the muscle activity recorded during MVICs and each set during the balance exercise were cut off. Afterwards, the mean sEMG amplitude was calculated for each muscle for the MVICs and exercise sets 1 to 3. The mean sEMG amplitude of the muscles during the balance exercise were normalized to their corresponding MVIC-sEMG values. Finally, the normalized sEMG data for synergistic muscles were averaged to provide an index of whole quadriceps (vastus medialis, rectus femoris, and vastus lateralis), hamstring (semitendinosus/semimembranosus and biceps femoris), tibialis (tibialis anterior), and triceps surae muscle activation (gastrocnemius medialis and soleus).

### Muscle oxygenation

During the experimental trials, changes in total tissue hemoglobin concentration (tHb) and oxygenated hemoglobin as a percentage of total hemoglobin (S_m_O_2_) were recorded and monitored using a mNIRS device (MOXY, Fortiori Design LLC, Hutchinson, MN, USA). Prior to the testing, the skinfold thickness of the vastus lateralis (13 ± 7 mm) was measured using a skinfold caliper (Harpenden Ltd., British Indicators Ltd, West Sussex, Great Britain) and the application area was shaved as well as cleaned with antiseptic solution. The MOXY device (61 × 44 × 21 mm, 48 g) was attached to the muscle belly of the vastus lateralis of the right thigh half distance between the patellar and the trochanter major. To avoid the influence of external light sources on data quality, a light protection rubber cap (125 mm diameter) was attached around the mNIRS device and fixed to the thigh with elastic adhesive tape. The location of the mNIRS device was marked to reproduce its application in the subsequent test sessions. S_m_O_2_ and tHb were recorded at rest in an upright standing position 60 s before the cuff was inflated (baseline) and throughout the exercise protocol.

The mNIRS data were recorded with a sampling rate of 2 Hz and filtered with a 4th order low-pass zero-phase Butterworth filter with a cutoff frequency of 0.2 Hz (Husmann et al. 2019). Data were averaged for the baseline time interval and for each set. With regard to data analysis, comparisons within conditions were performed using absolute values of S_m_O_2_ and tHb whereas percentage changes of S_m_O_2_ and tHb from baseline (∆S_m_O_2_ and ∆tHb) were used to interpret differences between conditions for each of the 3 sets.

### Ratings of effort perception and exercise-induced leg muscle pain

During the first laboratory visit, all participants were familiarized with the effort perception and exercise-induced leg muscle pain rating scales using standardized written descriptions (Husmann et al. 2019). The standardized instructions were additionally given at the beginning of each experimental condition. Effort perception and exercise-induced leg muscle pain were assessed using Borg’s 15-point perceived effort and pain scale, respectively (Borg 1982) and were quantified immediately after the termination of each set.

### Statistical analysis

Data analyses were conducted using JASP Statistics version 0.16.2 (University of Amsterdam, Amsterdam, Netherlands). Although data were checked for normality of distribution and homogeneity of variance using the Shapiro–Wilk and Levene’s tests, respectively, these results are not presented, since studies have shown that repeated measures analysis of variance (ANOVA) (Blanca et al. 2017) is stable against moderate violation of normality and homogeneity assumption, and therefore, nonparametric tests were not used to check for differences. Accordingly, three-way ANOVAs time × condition × sex with repeated measures were conducted for all data. If assumption of sphericity was violated, Greenhouse–Geisser correction was applied. All data were expressed as mean ± standard deviation (SD) and the level of significance was set at *p* ≤ 0.05. The effect size was determined by calculating partial eta squared (*η*_*p*_^*2*^), which was interpreted according to Cohen (2013) (0.01 = small, 0.06 = medium, and 0.14 = large effect). In case of significant main or interaction effects, post-hoc tests with Bonferroni correction were performed. Differences between (i.e., 80% AOP, 40% AOP, SHAM) and within conditions (i.e., for each time point) are presented as mean differences (MD) together with the 95% confidence intervals (95% CI). In addition, the effect size Cohen's *d* was calculated and interpreted using the following thresholds: *d* = 0.2 as a small, *d* = 0.5 as a medium, and *d* = 0.8 as a large effect (Cohen 2013).

## Results

All 24 participants successfully completed the three experimental sessions. Due to the low sample size and the unbalanced distribution of females using oral contraceptives (n = 7) and those not using hormonal preparations (n = 4) the statistical analyses of the influence of oral contraceptives and menstrual cycle related changes were not performed. However, the distribution of the phases within the menstrual cycle was equal between 80% and 40% AOP (menstruation = 4, follicular phase = 2, ovulation = 0, luteal phase = 5) while the SHAM condition was predominantly performed during the luteal phase (menstruation = 1, follicular phase = 3, ovulation = 0, luteal phase = 7). Furthermore, due to data loss because of measuring system errors, there were missing values for muscle activity of the quadriceps, triceps surae, tibialis (n = 1 [1f]), and hamstrings (n = 2 [1f/1 m]), as well as for sway distance and velocity (n = 2 [1f/1 m]). All statistical values of the non-significant interactions and main effects are presented in the supplemental material (Table S1).

### Maximal squat jump height

#### Fatigue development

A main effect of condition (*F* = 60.305, *p* < 0.001, *η*_*p*_^*2*^ = 0.733) was found for the decrease in squat jump performance. Post-hoc analysis revealed that the decline in maximal squat jump height was greater in the 80% AOP compared to the 40% AOP (MD = − 7.32% (95% CI − 9.86 to − 4.78%), *p* < 0.001, *d* = 1.80) and SHAM condition (MD = − 11.02% (95% CI − 13.56 to − 8.47%), *p* < 0.001, *d* = 2.71). Furthermore, the decline in maximal squat jump height was higher in the 40% AOP compared to the SHAM condition (MD = − 3.70% (95% CI − 6.24 to − 1.16%), *p* = 0.002, *d* = 0.91).

#### Recovery

**Fig. 2:**
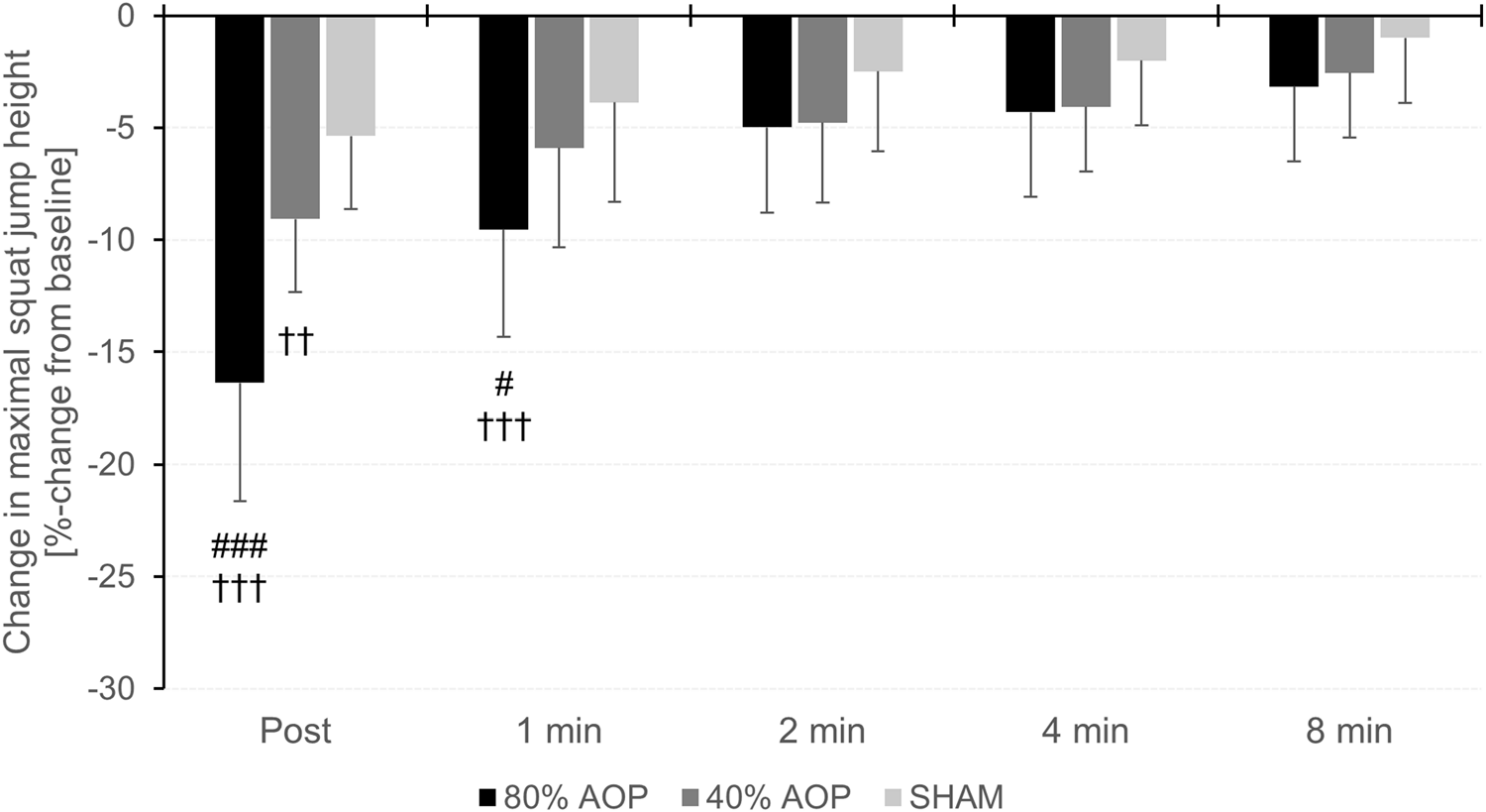
Decline in maximal squat jump height (post) and recovery of motor performance (1, 2, 4, and 8 min). Differences between conditions at specific time points (significant difference to 40% AOP: ^#^*p* < 0.05, ^##^*p* < 0.01, ^###^*p* < 0.001; significant difference to SHAM: ^†^*p* < 0.05, ^††^*p* < 0.01, ^†††^*p* < 0.001)

There was a time × condition interaction (*F* = 12.296, *p* < 0.001, *η*_*p*_^*2*^ = 0.359) as well as a main effect of time (*F* = 79.485, *p* < 0.001, *η*_*p*_^*2*^ = 0.783) and condition (*F* = 29.082, *p* < 0.001, *η*_*p*_^*2*^ = 0.569) for recovery of squat jump performance. Post-hoc tests showed that squat jump performance remained lower after 1 min in 80% AOP compared to 40% AOP (MD = − 3.63% (95% CI − 7.05 to − 0.22%), *p* = 0.022, *d* = 0.96) and SHAM (MD = − 5.66% (− 9.08 to − 2.24%), *p*  < 0.001, *d* = 1.49) while no difference was found between 40% AOP and SHAM (*p* = 1.000). There were no differences in maximal squat jump height between all three conditions after 2, 4, and 8 min post exercise (*p* = 1.000). The course of motor performance fatigue and recovery is shown in Fig. 2. Means ± SDs for squat jump height are given in Table 2.

**Table 2:** Motor performance fatigue and recovery (maximal squat jump height) as well as balance performance (sway distance, sway velocity), physiological responses (muscle activity, S_m_O_2_, tHb), and perceptual responses (ratings of effort perception, exercise-induced leg muscle pain) during the balance exercise in the different conditions (80% AOP, 40% AOP, SHAM). Data are expressed as means ± standard deviations

Parameter	Time point	Condition		
		80% AOP	40% AOP	SHAM
Maximal squat jump height (%-change from baseline)	Post 0 min	− 16.4 ± 5.2^###,†††^	− 9.1 ± 3.2^††^	− 5.4 ± 3.3
	Post 1 min	− 9.5 ± 4.8^##,†††^	− 5.9 ± 4.6	− 3.9 ± 4.4
	Post 2 min	− 5.0 ± 3.8	− 4.8 ± 2.8	− 2.5 ± 3.6
	Post 4 min	− 4.3 ± 3.8	− 4.1 ± 3.4	− 2.0 ± 2.9
	Post 8 min	− 3.2 ± 3.4	− 2.6 ± 3.1	− 1.0 ± 2.9
Balance performance				
Sway distance [cm]	Set 1	65.9 ± 14.8	64.9 ± 18.2	63.7 ± 15.9
	Set 2	65.6 ± 12.4	63.9 ± 13.4	64.3 ± 12.8
	Set 3	68.8 ± 12.2	61.7 ± 12.1	63.6 ± 12.4
Sway velocity [° · s^−1^]	Set 1	0.93 ± 0.20	0.91 ± 0.25	0.90 ± 0.23
	Set 2	0.93 ± 0.17	0.90 ± 0.19	0.91 ± 0.18
	Set 3	0.98 ± 0.19	0.87 ± 0.17	0.89 ± 0.17
Muscle activity [% of MVIC− sEMG]				
Quadriceps	Set 1	14.7 ± 5.4	14.1 ± 6.1	14.1 ± 4.5
	Set 2	15.6 ± 6.6	13.0 ± 5.3	12.8 ± 4.7
	Set 3	17.3 ± 7.9^###,†††^	13.1 ± 5.3	12.9 ± 4.8
Hamstrings	Set 1	7.4 ± 4.8	7.8 ± 5.4	6.9 ± 5.0
	Set 2	6.8 ± 4.6	7.4 ± 5.3	6.7 ± 4.6
	Set 3	6.9 ± 4.5	7.4 ± 5.7	6.8 ± 4.9
Tibialis	Set 1	11.6 ± 9.8	12.8 ± 9.7	17.7 ± 22.3
	Set 2	10.2 ± 9.5	9.0 ± 6.2	14.1 ± 18.8
	Set 3	10.4 ± 9.3	9.2 ± 6.4	14.3 ± 19.5
Triceps surae	Set 1	11.9 ± 5.3	15.4 ± 8.5	14.1 ± 7.9
	Set 2	10.8 ± 4.2	13.3 ± 6.7	13.0 ± 7.0
	Set 3	11.2 ± 3.6	12.9 ± 6.3	13.0 ± 7.5
S_m_O_2_ [%] (%-change from baseline)	Set 1	− 36.4 ± 23.4^###,†††^	− 14.6 ± 16.6^††^	5.6 ± 14.9
	Set 2	− 50.7 ± 26.6^###,†††^	− 14.9 ± 12.4^††^	5.8 ± 13.8
	Set 3	− 52.7 ± 23.2^###,†††^	− 17.9 ± 11.5^†††^	6.5 ± 14.6
tHb [a. u.] (%-change from baseline)	Set 1	3.2 ± 4.1	2.6 ± 4.9	1.8 ± 2.9
	Set 2	4.3 ± 4.0^††^	3.2 ± 5.1	2.1 ± 4.2
	Set 3	4.9 ± 4.2^†††^	3.6 ± 5.1	1.9 ± 4.0
Effort perception [a. u.]	Set 1	11.6 ± 2.4^##,†††^	10.6 ± 2.0	8.4 ± 1.9
	Set 2	13.8 ± 1.9^###,†††^	10.5 ± 2.3^†^	8.3 ± 2.1
	Set 3	15.5 ± 2.4^###,†††^	11.1 ± 2.6^†^	9.5 ± 2.4
Exercise-induced leg muscle pain [a. u.]	Set 1	12.3 ± 2.4^###,†††^	9.2 ± 2.3^†^	7.4 ± 1.8
	Set 2	14.5 ± 2.4^###,†††^	10.6 ± 2.5^††^	8.5 ± 2.3
	Set 3	16.4 ± 2.5^###,†††^	11.8 ± 2.8^†††^	9.0 ± 2.7

*AOP* arterial occlusion pressure; *a.u.* arbitrary unit; *sEMG* surface electromyography; *S*_*m*_*O*_*2*_ muscle oxygen saturation; *tHb* total hemoglobin

Differences between conditions at specific time points: significant difference to 40% AOP (^#^*p* < 0.05, ^##^*p* < 0.01, ^###^*p* < 0.001); significant difference to SHAM (^†^*p* < 0.05, ^††^*p* < 0.01, ^†††^*p *< 0.001)

### Balance performance

#### Total sway distance

A main effect of sex (*F* = 10.192, *p* = 0.005, *η*_*p*_^*2*^ = 0.338) was found for sway distance indicating that the total sway distance was higher in males compared to females across conditions and time points (MD = 13.37 cm (95% CI: 4.64 to 22.11 cm), *p* = 0.005, *d* = 1.08).

#### Sway velocity

**Fig. 3:**
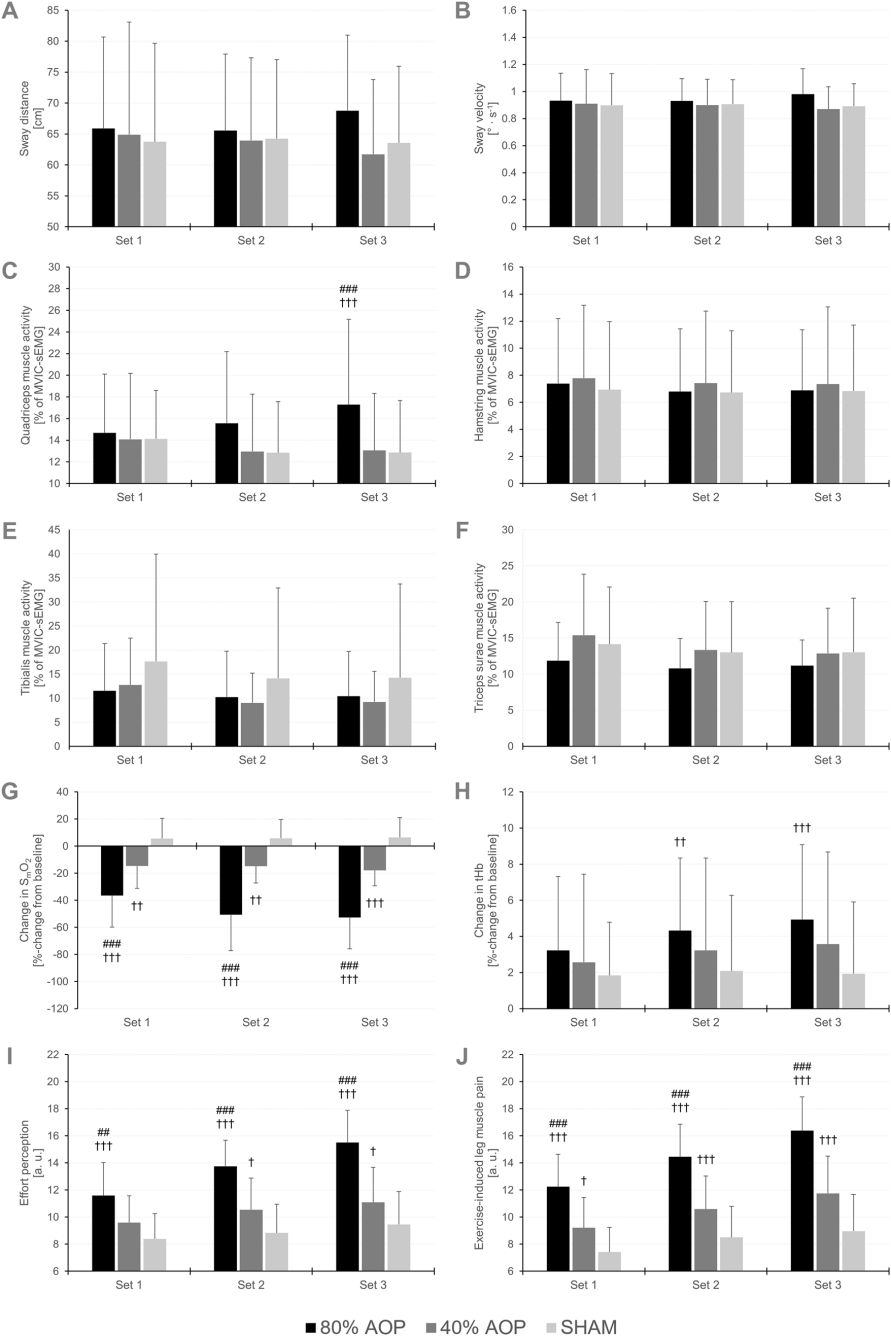
Balance performance (sway distance: **A**, sway velocity: **B**) as well as physiological (muscle activity (quadriceps: **C**, hamstrings: **D**, tibialis: **E**, triceps surae: **F**), muscle oxygen saturation [S_m_O_2_]: **G**, and total hemoglobin [tHb]: **H**), and perceptual measures (effort perception: **I**, exercise-induced leg muscle pain: **J**) during exercise (set 1–3). Differences between conditions at specific time points (significant difference to 40% AOP: ^#^*p* < 0.05, ^##^*p* < 0.01, ^###^*p* < 0.001; significant difference to SHAM: ^†^*p* < 0.05, ^††^*p* < 0.01, ^†††^*p* < 0.001)

There was a main effect of sex (*F* = 7.222, *p* = 0.014, *η*_*p*_^*2*^ = 0.265) for sway velocity revealing that sway velocity was higher in males compared to females across conditions and time points (MD = 0.16° · s^−1^ (95% CI: 0.04 to 0.29° · s^−1^), *p* = 0.014, *d* = 0.89). Sway distance and sway velocity during the balance exercise are shown in Fig. 3A-B. Descriptive data for balance performance are presented in Table 2.

### Muscle activity

#### Quadriceps

A time × condition (*F* = 12.829, *p* < 0.001, *η*_*p*_^*2*^ = 0.379), time × sex (*F* = 3.849, *p* = 0.044, *η*_*p*_^*2*^ = 0.155), and condition × sex interaction (*F* = 5.499, *p* = 0.008, *η*_*p*_^*2*^ = 0.208) as well as a main effect of condition (*F* = 7.021, *p* = 0.002, *η*_*p*_^*2*^ = 0.251) was found for quadriceps muscle activity. Post-hoc tests revealed that quadriceps muscle activity in set 3 was higher in the 80% AOP compared to 40% AOP condition (*MD* = 4.35% (95% CI 1.29 to 7.40%), *p* < 0.001, *d* = 0.80) and SHAM (*MD* = 4.83% (95% CI 1.77 to 7.89%), *p* < 0.001, *d* = 0.89). Furthermore, quadriceps muscle activity over the levels of time was higher in the 80% AOP condition compared to the SHAM condition in females (MD = 5.27% (95% CI 1.37 to 9.17%), *p* = 0.002, *d* = 0.97) but not in males (*p* = 1.000). However, there were no significant sex differences regarding time (*p* ≥ 0.135).

#### Hamstrings

There were no interactions or main effects for hamstring muscle activity.

#### Tibialis

There was a significant main effect of time (*F* = 6.791, *p* = 0.009, *η*_*p*_^*2*^ = 0.244) for tibialis muscle activity indicating a decrease from set 1 to set 2 (*MD* = − 2.76% (95% CI − 4.86 to − 0.66%), *p* = 0.006, *d* = 0.20) with set 3 lower than set 1 (MD = − 2.62% (95% CI − 4.72 to − 0.52%), *p* = 0.010, *d* = 0.19) but no difference between set 2 to set 3 (*p* = 1.000).

#### Triceps surae

A main effect of time (*F* = 11.014, *p* < 0.001, *η*_*p*_^*2*^ = 0.344) was found for triceps surae muscle activity. Post-hoc test showed a significant decrease from set 1 to set 2 (*MD* = − 1.38% (95% CI − 2.22 to − 0.53%), *p* < 0.001, *d* = 0.21) with set 3 lower than set 1 (MD = − 1.37% (95% CI − 2.21 to − 0.53%), *p*  < 0.001, *d* = 0.21) but no difference between set 2 to set 3 (*p* = 1.000). The course of muscle activity for the quadriceps, hamstrings, tibialis, and triceps surae during the balance exercise is shown in Fig. 3C-F. Means ± SDs for muscle activity are presented in Table 2.

### Muscle oxygenation

*∆S*_*m*_*O*_*2*_. A time × condition × sex (*F* = 7.323, *p* < 0.001, *η*_*p*_^*2*^ = 0.250), time × condition (*F* = 15.284, *p* < 0.001, *η*_*p*_^*2*^ = 0.410), and condition × sex interaction (*F* = 9.836, *p* = 0.002, *η*_*p*_^*2*^ = 0.309) as well as a main effect of time (*F* = 23.344, *p* < 0.001, *η*_*p*_^*2*^ = 0.515), condition (*F* = 70.926, *p* < 0.001, *η*_*p*_^*2*^ = 0.763), and sex (*F* = 19.233, *p* < 0.001, *η*_*p*_^*2*^ = 0.466) was found for ∆S_m_O_2_. Post-hoc analyses revealed that ∆S_m_O_2_ in males was highest in the 80% AOP condition in each set compared to the 40% AOP (set 1: MD = − 29.33% (95% CI − 52.91 to − 5.75%), *p* = 0.002, *d* = 1.95; set 2: MD = − 54.26% (95% CI − 77.84 to − 30.68%), *p* < 0.001, *d* = 3.61; set 3: MD = − 50.94% (95% CI − 74.52 to − 27.36%), *p* < 0.001, *d* = 3.39) and SHAM condition (set 1: MD = − 55.01% (95% CI − 78.59 to − 31.43%), *p* < 0.001, *d* = 3.67; set 2: MD = − 76.44% (95% CI − 100.02 to − 52.86%), *p* < 0.001, *d* = 5.08; set 3: MD = − 74.41% (95% CI − 97.99 to − 50.83%), *p*  < 0.001, *d* = 4.95), while ∆S_m_O_2_ in the 40% AOP condition was only higher in set 1 compared to the SHAM condition (MD = − 25.69% (95% CI − 49.27 to − 2.11%),  *p* = 0.016, *d* = 1.71) but not in set 2 (*p* = 0.103) and set 3 (*p* = 0.053). In females, ∆S_m_O_2_ was higher in the 80% AOP only compared to the SHAM condition during each set (set 1: MD = − 26.75% (95% CI 52.38 to − 1.11%), *p* = 0.029, *d* = 1.78; set 2: MD = − 32.85% (95% CI − 58.49 to − 7.22%), *p* = 0.001, *d* = 2.19; set 3: MD = − 41.06% (95% CI − 66.69 to − 15.42%), *p*  < 0.001, *d*  = 2.73). Regarding sex differences, ∆S_m_O_2_ was higher during each set in males only in the 80% AOP condition (set 1: MD = − 29.58% (95% CI − 52.71 to − 6.45%), *p*= 0.001, *d* = 1.97; set 2: MD = − 42.24% (95% CI − 65.37 to − 19.11%), *p* < 0.001, *d* = 2.81; set 3: MD = − 33.93% (95% CI − 57.06 to − 10.80%), *p* < 0.001, *d* = 2.26).

**Fig. 4:**
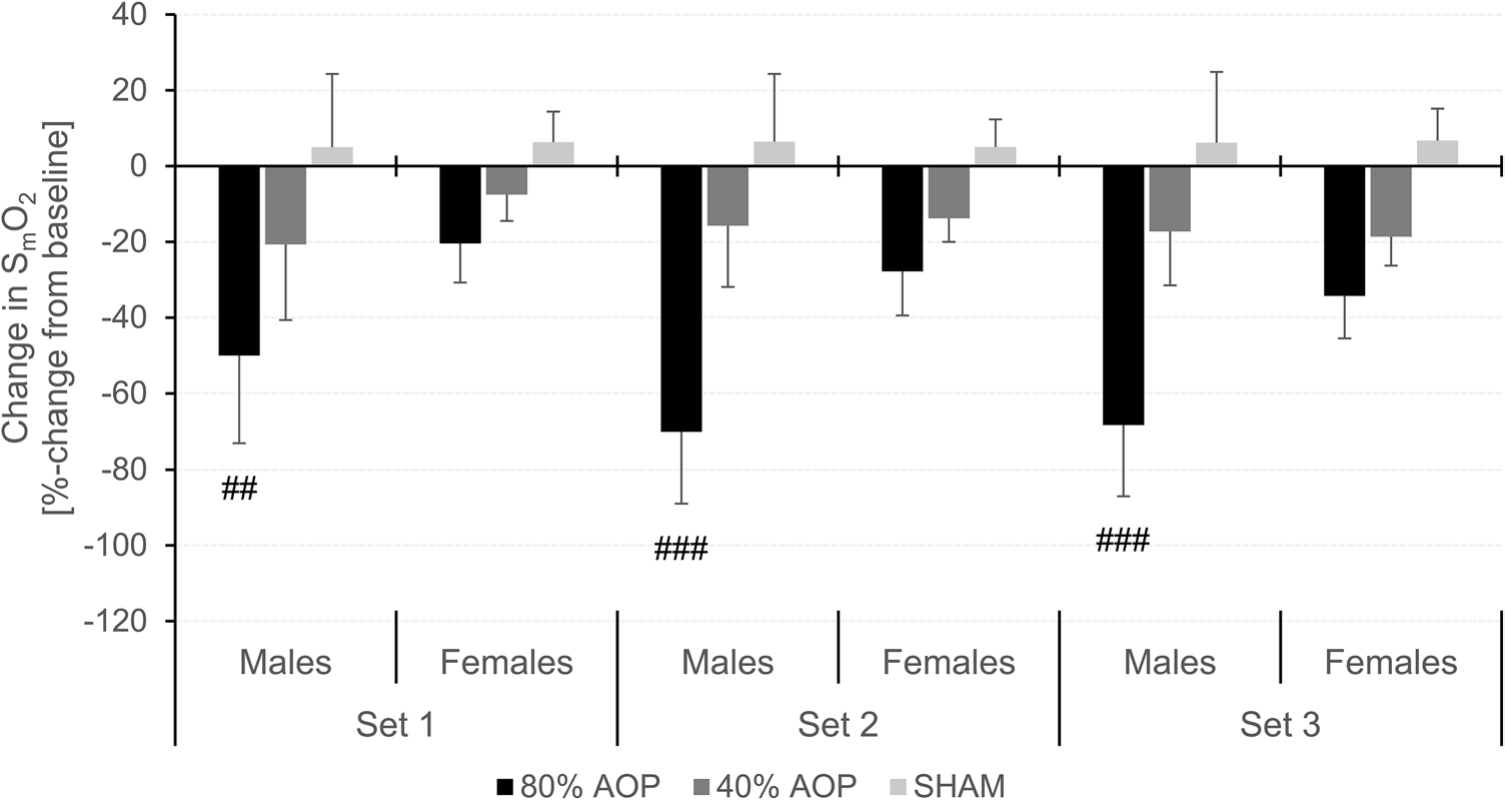
Comparison between males and females regarding changes in muscle oxygen saturation (S_m_O_2_) during exercise (set 1–3). Sex differences at specific time points within conditions (significant difference to females: ^#^*p* < 0.05, ^##^*p *< 0.01, ^###^*p* < 0.001)

*∆tHb*. A time × condition × sex (*F* = 8.936, *p* = 0.002, *η*_*p*_^*2*^ = 0.289) and a time × condition interaction (*F* = 17.407, *p* < 0.001, *η*_*p*_^*2*^ = 0.442) as well as a main effect of time (*F* = 37.872, *p* < 0.001, *η*_*p*_^*2*^ = 0.633) and condition (*F* = 8.444, *p* = 0.001, *η*_*p*_^*2*^ = 0.277) was found for ∆tHb. Post-hoc tests showed that ∆tHb was higher in set 2 and in set 3 compared to set 1 in the 80% AOP condition (MD = 1.66% (95% CI 0.76 to 2.55%), *p* < 0.001, *d* = 0.38 and MD = 2.46% (95% CI 1.57 to 3.35%), *p* < 0.001, *d* = 0.57, respectively) and in the 40% AOP condition (MD = 1.04% (95% CI 0.15 to 1.93%), *p* = 0.005, *d* = 0.24 and MD = 1.61% (95% CI 0.72 to 2.51%), *p* < 0.001, *d* = 0.37, respectively) but not in the SHAM condition (*p* = 1.000) in females. In males, only set 3 was higher compared to set 1 in the 80% AOP condition (MD = 1.07% (95% CI 0.25 to 1.89%), *p* < 0.001, *d* = 0.25). There were no further differences in ∆tHb (*p* ≥ 0.171). The course of ΔS_m_O_2_ and ΔtHb during the balance exercise is shown in Fig. 3G-H as well as split by sex in Fig. 4. Descriptive data for ΔS_m_O_2_ and ΔtHb are presented in Table 2.

### Ratings of effort perception and exercise-induced leg muscle pain

#### Effort perception

There was a significant time × condition interaction (*F* = 13.918, *p* < 0.001, *η*_*p*_^*2*^ = 0.387) as well as a main effect of time (*F* = 57.212, *p* < 0.001, *η*_*p*_^*2*^ = 0.722) and condition (*F* = 70.738, *p* < 0.001, *η*_*p*_^*2*^ = 0.763) for ratings of effort perception. Post-hoc tests revealed that ratings of effort perception were higher in each set in the 80% AOP compared to the 40% AOP condition (set 1: MD = 1.99 a. u. (95% CI 0.42 to 3.55 a. u.), *p* = 0.002, *d* = 0.88; set 2: MD = 3.16 a. u. (95% CI 1.60 to 4.72 a. u.), *p* < 0.001, *d* = 1.40; set 3: MD = 4.38 a. u. (95% CI 2.82 to 5.94 a. u.), *p* < 0.001, *d* = 1.94) and SHAM (set 1: MD = 3.24 a. u. (95% CI 1.68 to 4.80 a. u.), *p* < 0.001, *d* = 1.44; set 2: MD = 4.92 a. u. (95% CI 3.36 to 6.49 a. u.), *p* < 0.001, *d* = 2.18; set 3: MD = 6.06 a. u. (95% CI 4.50 to 7.62 a. u.), *p* < 0.001, *d* = 2.68). No differences in ratings of effort perception were found between the 40% AOP and SHAM condition in set 1 (*p* = 0.335). However, ratings of effort perception were higher in the 40% AOP compared to the SHAM condition in set 2 (*MD* = 1.77 a. u. (95% CI 0.20 to 3.33 a. u.), *p* = 0.012, *d* = 0.78) and set 3 (*MD* = 1.68 a. u. (95% CI 0.12 to 3.24 a. u.), *p* = 0.022, *d* = 0.74).

#### Exercise-induced leg muscle pain

There was a significant time × condition interaction (*F* = 11.088, *p* < 0.001, *η*_*p*_^*2*^ = 0.335) as well as a main effect of time (*F* = 95.749, *p* < 0.001, *η*_*p*_^*2*^ = 0.813) and condition (*F* = 107.747, *p* < 0.001, *η*_*p*_^*2*^ = 0.830). Post-hoc analysis showed that exercise-induced leg muscle pain was higher in the 80% AOP condition during each set compared to the 40% AOP condition (set 1: MD = 3.02 a. u. (95% CI 1.43 to 4.62 a. u.), *p* < 0.001, *d* = 1.26; set 2: MD = 3.82 a. u. (95% CI 2.23 to 5.42 a. u.), *p* < 0.001, *d* = 1.59; set 3: MD = 4.58 a. u. (95% CI 2.98 to 6.17 a. u.), *p* < 0.001, *d* = 1.91) and the SHAM condition (set 1: MD = 4.86 a. u. (95% CI 3.27 to 6.45 a. u.), *p* < 0.001, *d* = 2.02; set 2: MD = 6.01 a. u. (95% CI 4.42 to 7.61 a. u.), *p* < 0.001, *d* = 2.50; set 3: MD = 7.48 a. u. (95% CI 5.88 to 9.07 a. u.), *p* < 0.001, *d* = 3.11). Moreover, exercise-induced leg muscle pain in the 40% AOP condition was higher than in the SHAM condition in each set (set 1: MD = 1.84 a. u. (95% CI 0.24 to 3.43 a. u.), *p* = 0.010, *d* = 0.76; set 2: MD = 2.19 a. u. (95% CI 0.60 to 3.78 a. u.), *p* < 0.001, *d* = 0.91; set 3: MD = 2.90 a. u. (95% CI 1.30 to 4.49 a. u.), *p* < 0.001, *d* = 1.21). The course of ratings of effort perception and exercise-induced leg muscle pain during the balance exercise is shown in Fig. 3I-J. Means ± SDs are shown in Table 2.

## Discussion

The present study investigated the influence of three sets of a static balance exercise combined with different BFR pressures on motor performance fatigue and recovery as well as physiological and perceptual responses during exercise in males and females. The main findings were: (i) motor performance fatigue (i.e., decrease in squat jump height) was accelerated by both BFR pressures (80% and 40% AOP) compared to SHAM with a higher exercise-induced motor performance decrease in the high-pressure condition, (ii) the difference in motor performance fatigue between conditions was no longer evident at 2 min after exercise termination, (iii) sway distance and velocity remained constant over time while both were lower in females compared to males, (iv) muscle activity of the quadriceps during exercise only increased in the 80% AOP condition, (v) S_m_O_2_ decreased throughout the exercise in both 80% and 40% AOP conditions with the highest decrements in the high-pressure condition, while (vi) in the 80% AOP condition, the decrease in S_m_O_2_ during exercise was higher in males compared to females, and (vii) effort perception and exercise-induced leg muscle pain were higher in the BFR conditions compared to SHAM,

### Motor performance fatigue as well as physiological and perceptual responses

To the best of our knowledge, this is the first study demonstrating the influence of BFR on motor performance fatigue after three sets of a static balance exercise on an unstable surface. Both BFR conditions caused a greater decline in squat jump performance compared to the SHAM condition with the most pronounced decline in the high-pressure condition (80% AOP: − 16.4 ± 5.2%; 40% AOP: − 9.1 ± 3.2%; SHAM: − 5.4 ± 3.3%). Our results are in accordance with those of a study by Karabulut et al. (2010) who found a greater decrease in MVIC strength of the knee extensors after five sets of low-load resistance exercise (20 repetitions at 20% 1RM, 30 s rest between sets) with BFR (cuff pressure: 1.44 × systolic blood pressure; − 31%) compared to the same exercise protocol without BFR (− 13%). An explanation for the higher motor performance fatigue reported in the study by Karabulut et al. compared to our findings can be found in the differences between the exercise modalities (i.e., static double-leg stance on an unstable surface vs. unilateral leg extension exercise). Low-load resistance exercises are likely to require higher muscle forces compared to balance exercises resulting in an accelerated motor performance fatigue. Furthermore, the methods used to quantify motor performance fatigue differed between studies (squat jump height vs. MVIC strength of knee extensors).

The reduction in maximal motor performance during and after multiple sets of low-load resistance exercise with BFR can be mainly attributed to an accelerated decrease in the contractile function of muscles resulting in a reduced force-generating capacity (Husmann et al. 2018). Nevertheless, a decrease in neural drive to the muscles also plays a role, as it was shown in particular at the end of four sets (30-15-15-15 repetitions with 30 s rest in between) of low-load resistance exercise at 30% 1RM with a BFR pressure of 60% AOP (Husmann et al. 2018). Similar muscular and neural impairments can be assumed to have contributed to the decrease in maximal squat jump height after balance exercise with BFR in the present study. The main mechanisms responsible for the decline in contractile force of muscles may include the reduced Ca^2+^ release from the sarcoplasmic reticulum, the decreased myofibrillar Ca^2+^ sensitivity, and the impaired force-generating capacity of the cross-bridges per se (Cheng et al. 2018) as a result of the exacerbated accumulation of metabolites (e.g., inorganic phosphate, H^+^) (Cheng et al. 2018; Sugaya et al. 2011) due to the limited blood flow in both BFR conditions. However, metabolite accumulation during exercise cannot only impair contractile function but also the neural drive to muscles. Although various mechanisms have been discussed in this context (Gandevia 2001), it is thought that metabolite accumulation increases the inhibitory feedback from group III and IV muscle afferents, which, in turn, decreases motoneuron output (Butler et al. 2003) after BFR exercise (Husmann et al. 2018). The assumption of a higher BFR-induced metabolite accumulation is indirectly supported by the S_m_O_2_ data for the vastus lateralis, which were lower during balance exercise in both BFR conditions, and in particular in the 80% AOP condition, compared to SHAM.

That the drop in motor performance depends on the %AOP level was also shown by Dankel et al. (2017) who have investigated the effects of different %AOPs on MVIC strength of the elbow flexors after four sets of low-load resistance exercise (with 10%, 15%, and 20% 1RM). The authors have found that the high-pressure condition reduced MVIC strength to a larger extent than the low-pressure condition (80% AOP: − 17.0% vs. 40% AOP: − 5.2%). Therefore, it can be assumed that the accumulation of metabolites increases with higher %AOPs and was therefore responsible for the greater decline in maximal squat jump performance in the 80% AOP condition compared to the 40% AOP condition in the present study. Furthermore, the higher level of metabolic stress in the 80% AOP condition can be explained by the limited removal of metabolites during the rest intervals, thus leading to a higher reduction in contractile function and voluntary activation of muscles (Husmann et al. 2018; Dankel et al. 2017).

This is also supported by the finding that quadriceps muscle activity progressively increased in the 80% AOP condition, which is in line with the observation of a higher muscle activity during whole-body vibration combined with BFR compared to the same exercise without BFR (Centner et al. 2019). The increased muscle activity in the 80% AOP condition might be a mechanism to compensate for the decline in contractile function of muscles (Moritani et al. 1992) and to guarantee appropriate muscle forces for the execution of the balance task. Furthermore, a higher muscle activation during BFR exercise, as shown in the present study, is commonly thought to be associated with increased recruitment of type-II muscle fibers according to the Henneman’s size prinziple (Henneman et al. 1965), which might promote muscle hypertrophy (Pearson and Hussain 2015).

The effort perception data, which were higher in the 80% AOP compared to the SHAM condition, point to the same direction. Based on the corollary discharge model, perception of effort is generated by corollary discharges, which are associated with the central motor command (Pageaux 2016). Therefore, based on this model, it can be proposed that an increased motor command, which might have compensated for the contractile dysfunction in the BFR conditions, was responsible for the increased perception of effort. Furthermore, the BFR-induced stronger peripheral metabolic disturbances may have caused an increased afferent feedback from the working muscles (Proske and Gandevia 2012), which is also thought to contribute to the perception of effort (Pageaux 2016).

Exercise-induced leg muscle pain was also higher in both BFR conditions compared to SHAM, with even higher ratings in the 80% AOP condition compared to the 40% AOP condition. Higher BFR-induced pain ratings were also found during four sets (30-15-15-15 repetitions) of low-load knee extension exercise at 30% 1RM (Husmann et al. 2018). These data indicate an increased depolarization of nociceptors, which are sensitive to high amounts of metabolites associated with ischemic exercise (Jankowski et al. 2013). Furthermore, venous blood pooling induced by BFR exercise, especially with a high cuff pressure, might have contributed to the higher exercise-induced pain perception, given that Haouzi et al. (1999) have shown that venous expansion can stimulate group IV afferents. The higher exercise-induced pain ratings in the BFR-conditions might therefore serve as an indirect marker for the increased group III/IV muscle afferent feedback, which is also involved in the inhibition of the neural drive to muscles (Taylor et al. 2016).

Despite these differences in motor performance fatigue as well as the physiological and perceptual responses between the BFR conditions and SHAM as well as between the 80% AOP and 40% AOP condition, balance performance (i.e., sway distance, sway velocity) during the sets was not significantly affected. This finding is in agreement with the results of a recently published study showing that cycling at a power output corresponding to 40% of maximal oxygen uptake combined with BFR (80% AOP) for 20 min decreased drop jump performance, but not balance performance during perturbed stance (i.e., sway, maximal deviation) (Held et al. 2023). These findings collectively indicate that balance performance was not substantially influenced by the BFR-induced performance fatigue development and the physiological changes during exercise (i.e., quadriceps muscle activity and S_m_O_2_). These results may be of practical relevance, because people can benefit from the potential BFR-induced adaptations without increasing the risk of falling during balance exercise.

### Recovery of motor performance

Despite significant larger reductions in maximal motor performance directly after exercise in both BFR conditions, and in particular in the 80% AOP condition, motor performance recovered quickly and was not different between conditions already 2 min after exercise termination. More specifically, no differences in squat jump height between the SHAM condition and the 40% AOP as well as the 80% AOP condition were present 1 min and 2 min after exercise completion, respectively. These findings are in line with those of Husmann et al. (2018) who have investigated the acute impact of low-load resistance exercise with BFR on motor performance fatigue development and recovery of the knee extensors compared to non-restricted exercise. They have also found an accelerated motor performance fatigue development during and after BFR exercise, which was substantially recovered 2 min after exercise termination (Husmann et al. 2018). However, differences in motor performance disappeared after 1 min in the 40% AOP condition but not before 2 min in the 80% AOP condition compared to SHAM, respectively. This can be explained by the higher metabolite accumulation during the exercise in the 80% AOP condition, whose elimination probably required more time after cuff deflation. This may have led to prolonged impairments in contractile function due to changes in Ca^2+^ release or sensitivity.

Additionally, the longer performance reduction in the 80% AOP condition might also be a result of the higher metabolite-induced feedback from group III/IV muscle afferents affecting the excitability of spinal and supraspinal motoneurons, which contribute to a reduced neural drive to the muscles. However, the partial recovery of motor performance in the 80% AOP condition with no differences between all conditions 2 min after balance exercise is in line with the finding that not only the contractile function of muscles but also the neural impairments (i.e., decreased voluntary activation of muscles) quickly recover after low-load resistance exercise with BFR (Husmann et al. 2018).

### Sex differences

Males have shown a larger decrease in S_m_O_2_ (i.e., a higher ∆S_m_O_2_) in the 80% AOP condition in all three sets compared to females (see supplemental material [Table S2] and Fig. 4). This might be due to the higher density of capillaries per unit of muscle in the vastus lateralis in females compared to males due to a higher proportional type I muscle fiber area (Roepstorff et al. 2006). Moreover, it has been found that females have a greater vasodilatory response of feed arteries to the skeletal muscle, which might have promoted a higher muscle perfusion (Hunter 2014). These mechanisms might have slowed down the accumulation of metabolites. However, the decline in squat jump performance was similar for both sexes (see supplemental material [Table S2]) indicating that the better muscle oxygenation did not preserve neuromuscular function during this task. Therefore, other mechanisms might be responsible for the similar squat jump performance decline, which should be investigated by future studies. Although sway distance and velocity differed between sexes with lower values in females than in males, this might be related to differences in body weight and height, which is associated with the deviation and velocity of the center of pressure (Hue et al. 2007).

We have not found differences between females and males neither for muscle activity nor perceptual responses (i.e., ratings of effort perception and exercise-induced leg muscle pain). Of note, although S_m_O_2_, which represents the steady-state in oxygen supply and demand, was higher in females compared to males in the 80% AOP condition, there was no difference in exercise-induced leg muscle pain between sexes. This finding was surprising, given that S_m_O_2_ is a proxy for exercise intensity (Feldmann and Erlacher 2021) and the associated accumulation of metabolites. Theoretically, a higher S_m_O_2_ would therefore be associated with less accumulation of metabolites and a lower depolarization of group III/IV muscle afferents, which are thought to contribute to the perception of exercise-induced pain and the inhibition of spinal and/or supraspinal motoneurons (Hunter 2014). However, the underlying mechanism for this finding is not clear and requires further investigation.

Lastly, this study is not without limitations. A first limitation of the present study is that there was no implementation of a passive control group to verify if the decline in motor performance immediately after balance exercise was due to the exercise stimulus and not just a time-induced phenomenon. As a second limitation, the AOP was determined only during the familiarization session and only in the right leg. Although the laboratory visits were always at the same time of day and the participants were also instructed to reproduce their daily routine before each experimental trial, the AOP could have differed between trials and also between legs. However, a previous study by Hughes et al. (2021) has shown that there were no differences in AOP neither between the trials nor between legs. A third limitation is that the AOP was measured in a seated position, while the balance exercise was performed in a standing position. Therefore, the AOP in the balance exercise position might have been higher than the actually measured AOP with the participants sitting in an upright position (Hughes et al. 2018), which might have led to smaller relative BFR pressures than the specified 80% and 40% AOP. Lastly, in the present study, balance performance was not measured before and after the exercise without an inflated cuff (Held et al. 2023), but during the sets while wearing the inflated pneumatic cuff. Therefore, changes in balance performance might have been masked due to the cuff-induced mechanical alterations including a modification of muscle length due to the transversal pressure and the potential stabilization function for the hip joint induced by the inflated cuff.

## Conclusion

Our findings indicate, that static balance exercise combined with a high BFR pressure (i.e., 80% AOP) should provide the most effective stimulus to induce physiological and perceptual alterations, which might be associated with potential beneficial adaptations (e.g., muscle hypertrophy). The low BFR pressure (i.e., 40% AOP) might be not sufficient to induce comparable changes, when combined with three sets of a static balance exercise on an unstable surface. Therefore, balance exercise combined with a high BFR pressure might be an effective method for improving balance and strength at the same time. This might be of interest for populations who are not able to perform resistance training (e.g., patients after musculoskeletal injuries, frail elderly). Furthermore, despite the greater decline in motor performance after BFR exercise, balance performance was similar between all conditions indicating that balance performance was not significantly influenced by the pronounced motor performance fatigue in the high BFR pressure condition. These findings have relevance for practitioners in sports and rehabilitation settings as people may benefit from potential effects or adaptations induced by balance training with BFR without increasing the risk of falling. However, future research is required to investigate the chronic effects of balance training combined with BFR on the potential beneficial adaptations mentioned above.

### Supplementary Information

Below is the link to the electronic supplementary material.

Supplementary file1 (DOCX 24 KB)

Supplementary file2 (DOCX 33 KB)

## Data Availability

The datasets used and analyzed during the current study are available from the corresponding author on reasonable request.

## Abbreviations

ANOVA: Analysis of variance
AOP: Arterial occlusion pressure
BFR: Blood flow restriction
CI: Confidence interval
sEMG: surface Electromyography
MD: Mean difference
mNIRS: Muscular near-infrared spectroscopy
MVIC: Maximal voluntary isometric contraction
SD: Standard deviation
S_m_O_2_: Muscle oxygen saturation
THb: Total hemoglobin

## References

[CR1] Ansdell P, Brownstein CG, Škarabot J, Hicks KM, Howatson G, Thomas K, Hunter SK, Goodall S (2019). Sex differences in fatigability and recovery relative to the intensity-duration relationship. J Physiol.

[CR2] Ansdell P, Škarabot J, Atkinson E, Corden S, Tygart A, Hicks KM, Thomas K, Hunter SK, Howatson G, Goodall S (2020). Sex differences in fatigability following exercise normalised to the power-duration relationship. J Physiol.

[CR3] Behrens M, Mau-Moeller A, Weippert M, Fuhrmann J, Wegner K, Skripitz R, Bader R, Bruhn S (2015). Caffeine-induced increase in voluntary activation and strength of the quadriceps muscle during isometric, concentric and eccentric contractions. Sci Rep.

[CR4] Behrens M, Gube M, Chaabene H, Prieske O, Zenon A, Broscheid K-C, Schega L, Husmann F, Weippert M (2023). Fatigue and human performance: an updated framework. Sports Med.

[CR5] Bennett H, Slattery F (2019). Effects of Blood flow restriction training on aerobic capacity and performance: a systematic review. J Strength Cond Res.

[CR6] Biazon TMPC, Ugrinowitsch C, Soligon SD, Oliveira RM, Bergamasco JG, Borghi-Silva A, Libardi CA (2019). The association between muscle deoxygenation and muscle hypertrophy to blood flow restricted training performed at high and low loads. Front Physiol.

[CR7] Bielitzki R, Behrendt T, Behrens M, Schega L (2021). Current techniques used for practical blood flow restriction training: a systematic review. J Strength Cond Res.

[CR8] Blanca MJ, Alarcón R, Arnau J, Bono R, Bendayan R (2017). Non-normal data: Is ANOVA still a valid option?. Psicothema.

[CR9] Borg G (1982). Psychophysical bases of perceived exertion. Med Sci Sports Exerc.

[CR10] Butler JE, Taylor JL, Gandevia SC (2003). Responses of human motoneurons to corticospinal stimulation during maximal voluntary contractions and ischemia. J Neurosci.

[CR11] Centner C, Ritzmann R, Schur S, Gollhofer A, König D (2019). Blood flow restriction increases myoelectric activity and metabolic accumulation during whole-body vibration. Eur J Appl Physiol.

[CR12] Cheng AJ, Place N, Westerblad H (2018). Molecular basis for exercise-induced fatigue: the importance of strictly controlled cellular Ca2+ handling. Cold Spring Harb Perspect Med.

[CR13] Cohen J (2013). Statistical power analysis for the behavioral sciences.

[CR14] Cook DB, O'Connor PJ, Oliver SE, Lee Y (1998). Sex differences in naturally occurring leg muscle pain and exertion during maximal cycle ergometry. Int J Neurosci.

[CR15] Dankel SJ, Jessee MB, Buckner SL, Mouser JG, Mattocks KT, Loenneke JP (2017). Are higher blood flow restriction pressures more beneficial when lower loads are used?. Physiol Int.

[CR16] Enoka RM, Duchateau J (2016). Translating fatigue to human performance. Med Sci Sports Exerc.

[CR17] Feldmann A, Erlacher D (2021). Critical oxygenation: can muscle oxygenation inform us about critical power?. Med Hypotheses.

[CR18] Gandevia SC (2001). Spinal and supraspinal factors in human muscle fatigue. Physiol Rev.

[CR19] Gioftsidou A, Malliou P, Pafis G, Beneka A, Godolias G, Maganaris CN (2006). The effects of soccer training and timing of balance training on balance ability. Eur J Appl Physiol.

[CR20] Haouzi P, Hill JM, Lewis BK, Kaufman MP (1999). Responses of group III and IV muscle afferents to distension of the peripheral vascular bed. J Appl Physiol (bethesda, Md: 1985).

[CR21] Heitkamp HC, Horstmann T, Mayer F, Weller J, Dickhuth HH (2001). Gain in strength and muscular balance after balance training. Int J Sports Med.

[CR22] Held S, Rappelt L, Wiedenmann T, Deutsch J-P, Röttgen J, Donath L (2023). Blood flow restricted cycling impairs subsequent jumping but not balance performance slightly more than non-restricted cycling: an acute randomized controlled cross-over trial. J Sports Sci Med.

[CR23] Henneman E, Somjen G, Carpenter DO (1965). Excitability and inhibitability of motoneurons of different sizes. J Neurophysiol.

[CR24] Hue O, Simoneau M, Marcotte J, Berrigan F, Doré J, Marceau P, Marceau S, Tremblay A, Teasdale N (2007). Body weight is a strong predictor of postural stability. Gait Posture.

[CR25] Hughes L, Jeffries O, Waldron M, Rosenblatt B, Gissane C, Paton B, Patterson SD (2018). Influence and reliability of lower-limb arterial occlusion pressure at different body positions. PeerJ.

[CR26] Hughes L, Grant I, Patterson SD (2021). Aerobic exercise with blood flow restriction causes local and systemic hypoalgesia and increases circulating opioid and endocannabinoid levels. J Appl Physiol (bethesda, Md 1985).

[CR27] Hunter SK (2014). Sex differences in human fatigability: mechanisms and insight to physiological responses. Acta Physiol (oxf).

[CR28] Hunter SK (2018). Performance fatigability: mechanisms and task specificity. Cold Spring Harb Perspect Med.

[CR29] Husmann F, Mittlmeier T, Bruhn S, Zschorlich V, Behrens M (2018). Impact of blood flow restriction exercise on muscle fatigue development and recovery. Med Sci Sports Exerc.

[CR30] Husmann F, Bruhn S, Mittlmeier T, Zschorlich V, Behrens M (2019). Dietary nitrate supplementation improves exercise tolerance by reducing muscle fatigue and perceptual responses. Front Physiol.

[CR31] Iacovides S, Avidon I, Baker FC (2015). Does pain vary across the menstrual cycle? A Review. Eur J Pain.

[CR32] Jankowski MP, Rau KK, Ekmann KM, Anderson CE, Koerber HR (2013). Comprehensive phenotyping of group III and IV muscle afferents in mouse. J Neurophysiol.

[CR33] Jessee MB, Mattocks KT, Buckner SL, Dankel SJ, Mouser JG, Abe T, Loenneke JP (2018). Mechanisms of Blood Flow Restriction: The New Testament. Tech Orthop.

[CR34] Karabulut M, Cramer JT, Abe T, Sato Y, Bemben MG (2010). Neuromuscular fatigue following low-intensity dynamic exercise with externally applied vascular restriction. J Electromyogr Kinesiol.

[CR35] Lauver JD, Cayot TE, Rotarius T, Scheuermann BW (2017). The effect of eccentric exercise with blood flow restriction on neuromuscular activation, microvascular oxygenation, and the repeated bout effect. Eur J Appl Physiol.

[CR36] Lesinski M, Hortobágyi T, Muehlbauer T, Gollhofer A, Granacher U (2015). Dose-response relationships of balance training in healthy young adults: a systematic review and meta-analysis. Sports Medicine Auckland NZ.

[CR37] Lesinski M, Hortobágyi T, Muehlbauer T, Gollhofer A, Granacher U (2015). Effects of balance training on balance performance in healthy older adults: a systematic review and meta-analysis. Sports Med (auckland NZ).

[CR38] Loenneke JP, Fahs CA, Rossow LM, Sherk VD, Thiebaud RS, Abe T, Bemben DA, Bemben MG (2012). Effects of cuff width on arterial occlusion: implications for blood flow restricted exercise. Eur J Appl Physiol.

[CR39] Moritani T, Sherman WM, Shibata M, Matsumoto T, Shinohara M (1992). Oxygen availability and motor unit activity in humans. Europ J Appl Physiol.

[CR40] Mouser JG, Mattocks KT, Buckner SL, Dankel SJ, Jessee MB, Bell ZW, Abe T, Bentley JP, Loenneke JP (2019). High-pressure blood flow restriction with very low load resistance training results in peripheral vascular adaptations similar to heavy resistance training. Physiol Meas.

[CR41] Pageaux B (2016). Perception of effort in exercise science: definition, measurement and perspectives. Eur J Sport Sci.

[CR42] Pearson SJ, Hussain SR (2015). A review on the mechanisms of blood-flow restriction resistance training-induced muscle hypertrophy. Sports Med (auckland, NZ).

[CR43] Proske U, Gandevia SC (2012). The proprioceptive senses: their roles in signaling body shape, body position and movement, and muscle force. Physiol Rev.

[CR44] Roepstorff C, Thiele M, Hillig T, Pilegaard H, Richter EA, Wojtaszewski JFP, Kiens B (2006). Higher skeletal muscle alpha2AMPK activation and lower energy charge and fat oxidation in men than in women during submaximal exercise. J Physiol.

[CR45] Sugaya M, Yasuda T, Suga T, Okita K, Abe T (2011). Change in intramuscular inorganic phosphate during multiple sets of blood flow-restricted low-intensity exercise. Clin Physiol Funct Imaging.

[CR46] Taylor JL, Amann M, Duchateau J, Meeusen R, Rice CL (2016). Neural contributions to muscle fatigue: from the brain to the muscle and back again. Med Sci Sports Exerc.

[CR47] Wackerhage H, Schoenfeld BJ, Hamilton DL, Lehti M, Hulmi JJ (2019). Stimuli and sensors that initiate skeletal muscle hypertrophy following resistance exercise. J Appl Physiol (bethesda, Md.).

