# Supplementary material for: Acute effects of static balance exercise combined with different levels of blood flow restriction on motor performance fatigue as well as physiological and perceptual responses in young healthy males and females
Source: Eur J Appl Physiol. 2023 Jul 11;124(1):227–43. doi: 10.1007/s00421-023-05258-5 (PMC10787004; doi:10.1007/s00421-023-05258-5)
Supplement: Supplementary file 1 — Supplementary file1 (DOCX 24 KB) [file 421_2023_5258_MOESM1_ESM.docx]

**Supplemental material**

**Table S1**. Statistical values for the non-significant interactions and main effects in motor performance fatigue and recovery (maximal squat jump height) as well as balance performance (sway distance, sway velocity), muscle activity, muscle oxygenation (muscle oxygen saturation [S_m_O_2_], total tissue hemoglobin concentration [tHb]), and perceptual responses (ratings of effort perception, exercise-induced leg muscle pain). Results of significant interactions and main effects are not reported (denoted with "-").

|  | | **Interactions** | | | | **Main effects** | | |
| --- | --- | --- | --- | --- | --- | --- | --- | --- |
|  |  | **time × condition × sex** | **time × condition** | **time × sex** | **condition × sex** | **time** | **condition** | **sex** |
| **Maximal squat jump height** | **Fatigue development** | - | - | - | *F* = 1.027,  *p* = 0.342,  *η_p_^2^* = 0.045 | - | - | *F* = 0.035,  *p* = 0.854,  *η_p_^2^* = 0.002 |
|  | **Recovery** | *F* = 0.926,  *p* = 0.453,  *η_p_^2^* = 0.040 | - | *F* = 0.171,  *p* = 0.953,  *η_p_^2^* = 0.008 | *F* = 0.518,  *p* = 0.550,  *η_p_^2^* = 0.023 | - | - | *F* = 0.113,  *p* = 0.740,  *η_p_^2^* = 0.005 |
| **Balance performance** | **Sway distance** | *F* = 0.902,  *p* = 0.431,  *η_p_^2^* = 0.043 | *F* = 1.122,  *p* = 0.343,  *η_p_^2^* = 0.053 | *F* = 1.207,  *p* = 0.310,  *η_p_^2^* = 0.057 | *F* = 0.396,  *p* = 0.675,  *η_p_^2^* = 0.019 | *F* = 0.006,  *p* = 0.994,  *η_p_^2^* < 0.001 | *F* = 2.205,  *p* = 0.123,  *η_p_^2^* = 0.099 | - |
|  | **Sway velocity** | *F* = 0.413,  *p* = 0.178,  *η_p_^2^* = 0.020 | *F* = 1.045,  *p* = 0.373,  *η_p_^2^* = 0.050 | *F* = 0.941,  *p* = 0.399,  *η_p_^2^* = 0.045 | *F* = 0.467,  *p* = 0.630,  *η_p_^2^* = 0.023 | *F* = 0.014,  *p* = 0.986,  *η_p_^2^* < 0.001 | *F* = 2.779,  *p* = 0.074,  *η_p_^2^* = 0.122 | - |
| **Muscle activity** | **Quadriceps** | *F* = 2.133,  *p* = 0.113,  *η_p_^2^* = 0.092 | - | - | - | *F* = 3.149,  *p* = 0.071,  *η_p_^2^* = 0.130 | - | *F* = 3.059,  *p* = 0.095,  *η_p_^2^* = 0.127 |
|  | **Hamstrings** | *F* = 0.600,  *p* = 0.596,  *η_p_^2^* = 0.029 | *F* = 0.378,  *p* = 0.743,  *η_p_^2^* = 0.019 | *F* = 3.093,  *p* = 0.083,  *η_p_^2^* = 0.134 | *F* = 0.279,  *p* = 0.741,  *η_p_^2^* = 0.014 | *F* = 1.947,  *p* = 0.174,  *η_p_^2^* = 0.089 | *F* = 1.977,  *p* = 0.152,  *η_p_^2^* = 0.090 | *F* = 0.152,  *p* = 0.701,  *η_p_^2^* = 0.008 |
|  | **Tibialis** | *F* = 0.603,  *p* = 0.547,  *η_p_^2^* = 0.028 | *F* = 1.524,  *p* = 0.230,  *η_p_^2^* = 0.068 | *F* = 0.438,  *p* = 0.571,  *η_p_^2^* = 0.020 | *F* = 0.522,  *p* = 0.507,  *η_p_^2^* = 0.024 | - | *F* = 2.185,  *p* = 0.149,  *η_p_^2^* = 0.094 | *F* = 0.746,  *p* = 0.397,  *η_p_^2^* = 0.034 |
|  | **Triceps surae** | *F* = 0.542,  *p* = 0.589,  *η_p_^2^* = 0.025 | *F* = 1.692,  *p* = 0.196,  *η_p_^2^* = 0.075 | *F* = 1.271,  *p* = 0.286,  *η_p_^2^* = 0.057 | *F* = 1.761,  *p* = 0.189,  *η_p_^2^* = 0.077 | - | *F* = 2.843,  *p* = 0.069,  *η_p_^2^* = 0.119 | *F* = 0.118,  *p* = 0.735,  *η_p_^2^* = 0.006 |
| **Muscle oxygenation** | **∆S_m_O_2_** | - | - | *F* = 2.089,  *p* = 0.136,  *η_p_^2^* = 0.087 | - | - | - | - |
|  | **∆tHb** | - | - | *F* = 3.589,  *p* = 0.067,  *η_p_^2^* = 0.140 | *F* = 1.232,  *p* = 0.301,  *η_p_^2^* = 0.053 | - | - | *F* = 0.752,  *p* = 0.395,  *η_p_^2^* = 0.033 |
| **Perceptual ratings** | **Effort perception** | *F* = 0.324,  *p* = 0.737,  *η_p_^2^* = 0.015 | - | *F* = 0.293,  *p* = 0.660,  *η_p_^2^* = 0.013 | *F* = 1.295,  *p* = 0.284,  *η_p_^2^* = 0.056 | - | - | *F* = 0.403,  *p* = 0.532,  *η_p_^2^* = 0.018 |
|  | **Exercise-induced leg muscle pain** | *F* = 1.196,  *p* = 0.315,  *η_p_^2^* = 0.052 | - | *F* = 0.029,  *p* = 0.907,  *η_p_^2^* = 0.001 | *F* = 2.977,  *p* = 0.061,  *η_p_^2^* = 0.119 | - | - | *F* = 0.431,  *p* = 0.518,  *η_p_^2^* = 0.019 |
