# Supplementary material for: Acute effects of static balance exercise combined with different levels of blood flow restriction on motor performance fatigue as well as physiological and perceptual responses in young healthy males and females
Source: Eur J Appl Physiol. 2023 Jul 11;124(1):227–43. doi: 10.1007/s00421-023-05258-5 (PMC10787004; doi:10.1007/s00421-023-05258-5)
Supplement: Supplementary file 2 — Supplementary file2 (DOCX 33 KB) [file 421_2023_5258_MOESM2_ESM.docx]

**Supplemental Material**

**Table S2**. Sex differences in motor performance fatigue and recovery (maximal squat jump height) as well as balance performance (sway distance, sway velocity), physiological responses (muscle activity, S_m_O_2_, tHb), and perceptual responses (ratings of effort perception, exercise-induced leg muscle pain) during the balance exercise in the different conditions (80% AOP, 40% AOP, SHAM). Data are expressed as means ± standard deviations.

| **Parameter** | | **Time Point** | **Condition** | | | | | |
| --- | --- | --- | --- | --- | --- | --- | --- | --- |
|  |  |  | **80% AOP** | | **40% AOP** | | **SHAM** | |
| **Sex** | |  | **Male** | **Female** | **Male** | **Female** | **Male** | **Female** |
| **Maximal squat jump height**  **(%-change from baseline)** | | **Post 0 min** | -15.2 ± 4.9 | -17.8 ± 5.5 | -9.3 ± 3.4 | -8.8 ± 3.1 | -5.5 ± 3.4 | -5.2 ± 3.3 |
|  |  | **Post 1 min** | -9.2 ± 4.06 | -10.0 ± 5.7 | -5.3 ± 4.4 | -6.7 ± 5.0 | -3.2 ± 4.9 | -4.7 ± 3.9 |
|  |  | **Post 2 min** | -5.7 ± 3.4 | -4.2 ± 4.3 | -4.3 ± 3.0 | -5.7 ± 2.5 | -2.5 ± 4.0 | -2.5 ± 3.1 |
|  |  | **Post 4 min** | -4.2 ± 3.3 | -4.4 ± 4.4 | -3.7 ± 3.9 | -4.5 ± 2.9 | -1.9 ± 3.0 | -2.2 ± 2.9 |
|  |  | **Post 8 min** | -1.8 ± 2.0 | -4.7 ± 4.1 | -2.5 ± 3.2 | -2.6 ± 3.2 | -1.2 ± 2.5 | -0.8 ± 3.5 |
| **Balance performance** | Sway distance [cm] | **Set 1** | 71.8 ± 15.3 | 58.8 ± 10.9 | 74.4 ± 16.3^#^ | 53.5 ± 13.6 | 69.5 ± 15.3 | 56.8 ± 14.4 |
|  |  | **Set 2** | 71.2 ± 12.2 | 58.8 ± 9.0 | 69.7 ± 12.1 | 57.0 ± 11.9 | 69.8 ± 12.3 | 57.6 ± 10.3 |
|  |  | **Set 3** | 73.6 ± 10.3 | 63.0 ± 12.2 | 67.0 ± 10.7 | 55.4 ± 10.8 | 70.1 ± 8.4 | 55.8 ± 12.2 |
|  | Sway velocity [° · s^-1^] | **Set 1** | 1.00 ± 0.2 | 0.85 ± 0.16 | 1.02 ± 0.24 | 0.76 ± 0.18 | 0.97 ± 0.23 | 0.82 ± 0.21 |
|  |  | **Set 2** | 1.01 ± 0.16 | 0.85 ± 0.14 | 0.98 ± 0.17 | 0.79 ± 0.17 | 0.98 ± 0.17 | 0.82 ± 0.15 |
|  |  | **Set 3** | 1.03 ± 0.15 | 0.92 ± 0.21 | 0.93 ± 0.14 | 0.78 ± 0.15 | 0.96 ± 0.13 | 0.82 ± 0.16 |

| **Muscle activity**  **[% of MVC-EMG]** | Quadriceps | **Set 1** | 13.2 ± 3.6 | 16.7 ± 6.9 | 11.8 ± 3.0 | 17.1 ± 7.8 | 14.3 ± 3.7 | 13.9 ± 5.5 |
| --- | --- | --- | --- | --- | --- | --- | --- | --- |
|  |  | **Set 2** | 13.5 ± 4.3 | 18.3 ± 8.2 | 10.9 ± 2.8 | 15.7 ± 6.6 | 12.6 ± 4.7 | 13.2 ±5.0 |
|  |  | **Set 3** | 14.3 ± 4.6 | 21.2 ± 9.7 | 10.8 ± 2.8 | 16.0 ± 6.3 | 12.5 ± 4.3 | 13.3 ± 5.6 |
|  | Hamstrings | **Set 1** | 7.4 ± 5.5 | 7.4 ± 4.2 | 6.6 ± 4.2 | 9.2 ± 6.5 | 7.9 ± 6.0 | 5.8 ± 3.5 |
|  |  | **Set 2** | 6.6 ± 4.9 | 7.0 ± 4.5 | 6.1 ± 3.9 | 9.0 ± 6.5 | 7.5 ± 5.4 | 5.8 ± 3.4 |
|  |  | **Set 3** | 6.3 ± 4.7 | 7.6 ± 4.3 | 5.7 ± 3.9 | 9.4 ± 7.0 | 7.6 ± 5.9 | 5.9 ± 3.3 |
|  | Tibialis | **Set 1** | 10.0 ± 8.6 | 13.6 ± 11.4 | 12.9 ± 11.4 | 12.5 ± 7.7 | 15.0 ± 20.1 | 21.1 ± 25.5 |
|  |  | **Set 2** | 8.6 ± 7.4 | 12.4 ± 11.8 | 8.3 ± 7.0 | 10.0 ± 5.2 | 10.5 ± 9.9 | 18.8 ± 26.2 |
|  |  | **Set 3** | 8.3 ± 7.6 | 13.2 ± 10.9 | 8.5 ± 7.7 | 10.2 ± 4.4 | 11.6 ± 14.9 | 17.7 ± 24.7 |
|  | Triceps surae | **Set 1** | 12.0 ± 4.9 | 11.6 ± 6.1 | 16.0 ± 5.6 | 14.6 ± 11.5 | 13.0 ± 5.6 | 15.6 ± 10.4 |
|  |  | **Set 2** | 10.7 ± 3.4 | 10.9 ± 5.2 | 14.0 ± 4.3 | 12.5 ± 9.2 | 11.4 ± 4.0 | 15.2 ± 9.5 |
|  |  | **Set 3** | 10.6 ± 2.6 | 11.9 ± 4.5 | 13.1 ± 4.5 | 12.6 ± 8.3 | 11.7 ± 5.4 | 14.8 ± 10.6 |
| **S_m_O_2_**  **(% change from baseline)** | | **Set 1** | -50.0 ± 23.0^##^ | -20.4 ± 10.3 | -20.7 ± 20.0 | -7.5 ± 7.0 | 5.0 ± 19.3 | 6.3 ± 8.0 |
|  |  | **Set 2** | -70.0 ± 19.0^###^ | -27.8 ± 11.7 | -15.8 ± 16.2 | -13.8 ± 6.2 | 6.4 ± 17.9 | 5.1 ± 7.2 |
|  |  | **Set 3** | -68.2 ± 18.8^###^ | -34.3 ± 11.2 | -17.3 ± 14.2 | -18.6 ± 7.7 | 6.2 ± 18.7 | 6.8 ± 8.4 |
| **tHb**  **[a. u.] (% change from baseline)** | | **Set 1** | 4.2 ± 5.2 | 2.1 ± 1.9 | 3.2 ± 6.7 | 1.9 ± 0.7 | 2.7 ± 3.6 | 0.8 ± 1.4 |
|  |  | **Set 2** | 4.8 ± 5.2 | 3.8 ± 1.9 | 3.5 ± 7.0 | 2.9 ± 0.7 | 3.3 ± 5.4 | 0.7 ± 1.4 |
|  |  | **Set 3** | 5.2 ± 5.5 | 4.6 ± 1.9 | 3.7 ± 7.0 | 3.5 ± 0.9 | 3.1 ± 5.0 | 0.5 ± 1.4 |
| **Effort perception [a. u.]** | | **Set 1** | 11.4 ± 2.2 | 11.8 ± 2.8 | 9.2 ± 1.9 | 10.0 ± 2.1 | 8.5 ± 1.9 | 8.2 ± 2.0 |
|  |  | **Set 2** | 13.7 ± 2.1 | 13.8 ± 1.8 | 9.9 ± 2.4 | 11.3 ± 2.2 | 8.9 ± 2.2 | 8.8 ± 2.2 |
|  |  | **Set 3** | 15.3 ± 2.5 | 15.7 ± 2.3 | 10.5 ± 2.7 | 11.8 ± 2.4 | 9.5 ± 2.4 | 9.5 ± 2.5 |
| **Exercise-induced leg muscle pain [a. u.]** | | **Set 1** | 12.0 ± 2.4 | 12.6 ± 2.5 | 8.8 ± 2.0 | 9.7 ± 2.5 | 7.5 ± 1.7 | 7.4 ± 2.1 |
|  |  | **Set 2** | 14.2 ± 2.8 | 15.7 ± 2.0 | 9.8 ± 2.2 | 11.6 ± 2.5 | 8.9 ± 2.3 | 8.1 ± 2.3 |
|  |  | **Set 3** | 16.1 ± 2.7 | 16.7 ± 2.3 | 10.0 ± 2.7 | 12.7 ± 2.6 | 9.3 ± 2.9 | 8.6 ± 2.6 |
| AOP, arterial occlusion pressure; a.u., arbitrary unit; EMG, electromyography; S_m_O_2_, muscle oxygen saturation; tHb, total hemoglobin | | | | | | | | |

Differences between males and females at specific time points: significant difference to females (^##^p < 0.01, ^###^p < 0.001)
